# Investigating heterogeneities of live mesenchymal stromal cells using AI-based label-free imaging

**DOI:** 10.1038/s41598-021-85905-z

**Published:** 2021-03-24

**Authors:** Sara Imboden, Xuanqing Liu, Brandon S. Lee, Marie C. Payne, Cho-Jui Hsieh, Neil Y. C. Lin

**Affiliations:** 1 Department of Mechanical and Aerospace Engineering, University of California, Los Angeles, 90095 USA; 2 Department of Computer Science, University of California, Los Angeles, 90095 USA; 3 Department of Bioengineering, University of California, Los Angeles, 90095 USA; 4 Institute for Quantitative and Computational Biosciences, University of California, Los Angeles, 90095 USA

**Keywords:** Biotechnology, Cell biology, Stem cells, Computational biology and bioinformatics, Machine learning

## Abstract

Mesenchymal stromal cells (MSCs) are multipotent cells that have great potential for regenerative medicine, tissue repair, and immunotherapy. Unfortunately, the outcomes of MSC-based research and therapies can be highly inconsistent and difficult to reproduce, largely due to the inherently significant heterogeneity in MSCs, which has not been well investigated. To quantify cell heterogeneity, a standard approach is to measure marker expression on the protein level via immunochemistry assays. Performing such measurements non-invasively and at scale has remained challenging as conventional methods such as flow cytometry and immunofluorescence microscopy typically require cell fixation and laborious sample preparation. Here, we developed an artificial intelligence (AI)-based method that converts transmitted light microscopy images of MSCs into quantitative measurements of protein expression levels. By training a U-Net+ conditional generative adversarial network (cGAN) model that accurately (mean $r_s$ = 0.77) predicts expression of 8 MSC-specific markers, we showed that expression of surface markers provides a heterogeneity characterization that is complementary to conventional cell-level morphological analyses. Using this label-free imaging method, we also observed a multi-marker temporal-spatial fluctuation of protein distributions in live MSCs. These demonstrations suggest that our AI-based microscopy can be utilized to perform quantitative, non-invasive, single-cell, and multi-marker characterizations of heterogeneous live MSC culture. Our method provides a foundational step toward the instant integrative assessment of MSC properties, which is critical for high-throughput screening and quality control in cellular therapies.

## Introduction

Mesenchymal stromal cell (MSC) therapy offers a promising treatment option for inflammatory disorders^1^, immune-mediated diseases^2^, and neurological damages^3^. MSCs are popular for their easy in vitro proliferation^4^ and ability to differentiate into various mesenchymal tissues^4,5^. MSC tissue culture has been shown to modulate immune responses for autoimmune diseases, sepsis, and transplant surgery^6–9^. The efficacy of these critical therapeutic functions is difficult to predict due to the inherent MSC heterogeneity, arising from variations between MSC donors, batches, and clones^10–13^. Understanding and controlling this notorious cellular heterogeneity in vitro has been a central challenge in both basic and translational research^14,15^. Unfortunately, it has been difficult to precisely define and measure such heterogeneity in live MSCs, since current MSC characterization methods are either relatively non-specific, time-consuming, or invasive^16,17^.

Traditional MSC characterization methods have focused on analyzing either the morphological phenotypes^18–23^ or expression of surface markers^24–27^. While measuring cellular morphology allows for non-invasive assessment of live cells, no strong scientific link has been established between cellular morphology and MSC characteristics. In contrast, molecular-based gene expression measurements, which primarily rely on immunochemistry assays for quantifying protein expression, provide more rigorous analysis of MSC characteristics^28^. The downside of these assays is that they usually require laborious sample preparation, damaging cell fixation, and repetitive experimental procedures for multiple marker analysis^29,30^. Collectively, the inability to perform non-invasive, instant, and biologically rigorous characterizations substantially limit the therapeutic potential of MSCs.

To address these technical challenges, we developed an AI-based label-free imaging platform for studying the characteristic heterogeneity in live MSCs. AI-based image translation approaches have been proven to be useful tools for visualizing 3D organelle structures^30^, distinguishing cell types and states^31,32^, and improving image quality^33–36^, segmentation^37^, and restoration^38^.

In this work, we developed a deep convolutional neural network (CNN) to predict immunofluorescent-like images using transmitted light microscopy data. This non-invasive imaging method allows us to directly observe gene expression on the protein level for multiple markers simultaneously in live MSCs. We demonstrated that the AI-translated immunofluorescence images showed a high degree of similarity to the ground truth and can be applied to quantitative studies. Using the results of our trained model, we combined gene expression levels of 8 common MSC markers with the measurement of 12 morphological features to draw deeper conclusions about MSC heterogeneity. From clustering and PCA analysis we found that while both morphology and gene expression can be used to effectively identify MSC heterogeneities, they provide contrasting assessments on cell characteristics. Lastly, utilizing the AI-predicted images, we performed further analyses to profile gene expression fluctuations and characterize protein localizations on the sub-cellular level. Our model has potential to advance current methods for assessing cellular heterogeneity and can be broadly applied in clinical and therapeutic applications.

## Results

### Machine learning model development

**Figure 1:**
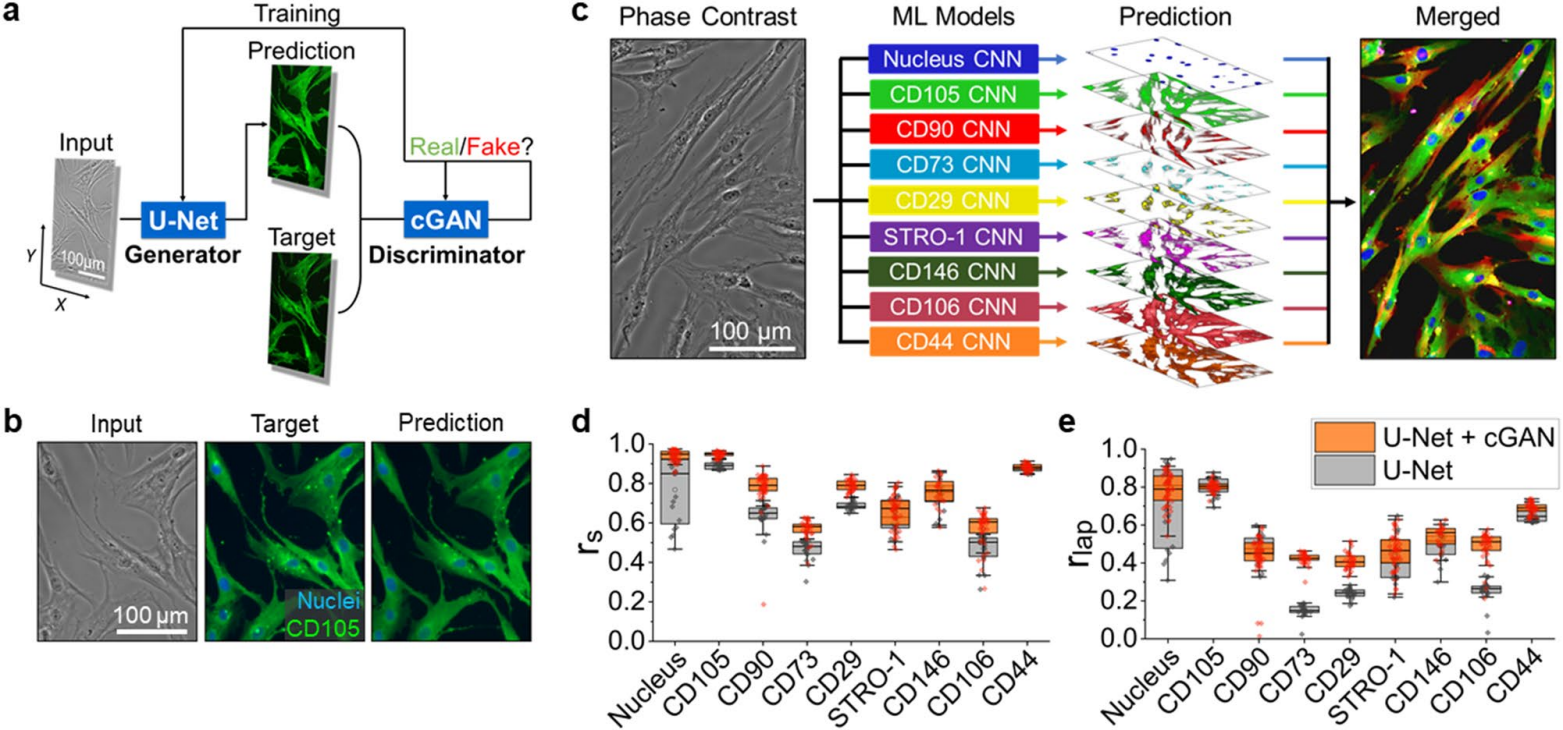
Convolutional neural network (CNN) training for predicting marker expression. (**a**) Schematic of an iterative machine learning training operation. The generator produces a virtually stained prediction image using a U-Net architecture. The discriminator measures the probability of similarity between prediction and target and updates loss function parameters. (**b**) Left to right: example of a phase contrast input image, a target (ground truth) immunofluorescence image and the respective prediction image. (**c**) Individually trained marker predictions from a single transmitted light microscopy image combined into a multi-marker composite. (**d**, **e**) Performance of virtual surface marker prediction using the U-Net + cGAN model (orange) versus the U-Net only model (grey). Both (**d**), the Source Pearson correlation coefficient $r_{s}$, and (**e**), the Laplacian Pearson correlation coefficient $r_{lap}$, demonstrate higher values over all channels. Each data point represents one target-prediction image pair, with outliers shown as diamonds. Boxplots were created by using OriginLab version 2019.

Our machine learning (ML) model pairs two convolutional neural networks (CNNs), one a generator and the other a discriminator (Fig. 1a). The generator network, based on a U-Net architecture (Fig. S1)^30,39^, learns the nonlinear relationship between the phase contrast image and its corresponding fluorescent target. During training, the neural network minimizes the loss function that quantifies the pixel-to-pixel differences between the predicted and target image. Here, the target is a fluorescent image of cultured MSCs at passage 3 that are immunofluorescently labeled (“Methods”). After propagating through the U-Net, the resultant image is loaded into the discriminator network, developed using a conditional generative adversarial network (cGAN) (Fig. S1)^40^, which evaluates the probability of similarity between prediction and target. During training, the discriminator output, an adaptive loss function, is iterated over a set number of cycles through the model to optimize the prediction. Following the completion of the iteration process, the resultant trained model was used for predicting virtual fluorescent MSC labels from unused phase contrast data.

We found that the trained AI model is able to predict images that closely recapitulate many features found in the target on multiple length scales. An example of model prediction is shown in Fig. 1b where the target is a fluorescent image of MSCs stained for CD105, a membrane glycoprotein that has been commonly used as a positive MSC marker. As demonstrated in the figure, the prediction captured the overall intensity distribution, cell morphology, and sub-cellular structures including protein localization and nucleus shape.

One advantage of our machine learning approach is the individual training process for each marker, allowing us to avoid emission channel cross-contamination in multi-color imaging and improve the fluorescent signal specificity. Moreover, since individual models can be directly combined upon completion of training, there is no limit on the number of markers that can be predicted simultaneously from one phase contrast input. Applying this feature, we trained a panel of genes comprising 8 MSC markers, which were strategically selected to cover a wide range of MSC properties; the markers included CD105, CD90, and CD73 to define MSC subpopulations^41^, CD29 to implicate cell migration^42^, CD146 to show vascular smooth muscle commitment^43^, CD106 (VCAM1)^44^ and STRO-1^45^ to illustrate MSC immunomodulation capacity, and CD44 to designate the cell-cell and cell-matrix interactions^46^. We show the combined predicted image composite in Fig. 1c, in which each marker exhibits distinct distributions and local enrichments within the cells. We have shown that this approach can also be used to process stitched tile images (Fig. S2).

To evaluate the prediction accuracy of our model, we calculated the pixel-level Pearson correlation coefficient $r_s$ (“Methods”) between the prediction and target images. Overall, we observed fairly high accuracy in all tested markers with an $r_s$ average of $\sim$ 0.77 (Fig. 1d). However, the actual prediction accuracy depends on the specific marker of interest. For example, the surface proteins that show a more uniform distribution (e.g., CD105 and CD44) exhibit higher values of $r_s$ than the markers that show more protein localization (e.g., CD73 and CD29). By comparing our results with the data from a generator-only model (i.e., U-Net only), we found that the addition of a discriminator CNN improved both the prediction accuracy and robustness (Fig. S3) for most tested markers. Such improvements were also found in the Laplacian Pearson correlation analysis $r_{lap}$ (Fig. S4, “Methods”) that compares the Laplacian fields of prediction and target images (Fig. 1e). Here, the real-space analysis $r_s$ focuses on the overall intensity distribution, whereas the Laplacian-space analysis $r_{lap}$ highlights the details of signal variation. This prediction accuracy evaluation was repeated using a different measurement metric (absolute error)^47^ and consistent results were obtained (Fig. S5).

### Characterizations of predicted images

To characterize the robustness of our ML model, we studied how training data properties including dataset size, signal-to-noise ratio (SNR) of the target images, and the presence of impurities, influence the prediction accuracy. For simplicity, we focused on CD105 as it shows the highest Pearson correlation coefficient, allowing us to perturb the training data systematically.

**Figure 2:**
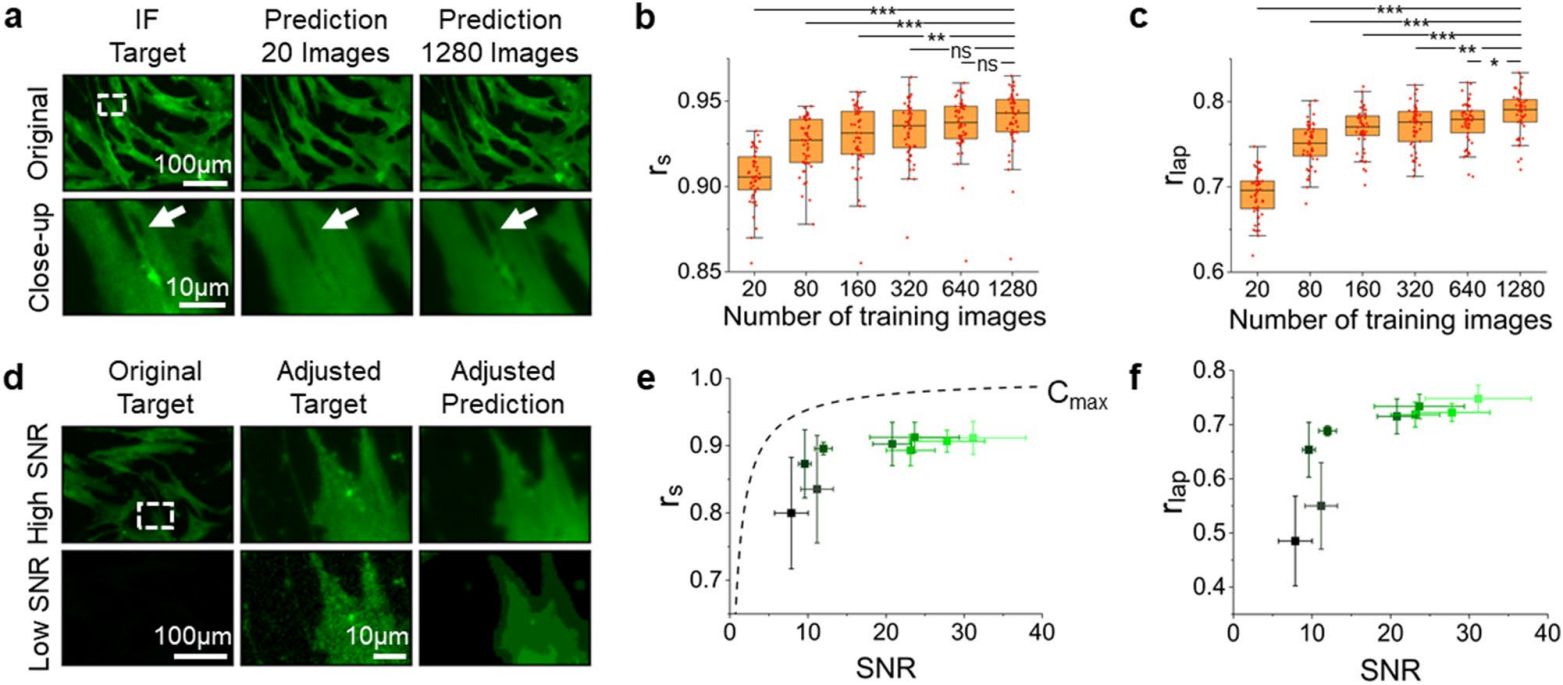
Characterization and robustness analysis of the proposed ML model with CD105 staining. (**a**) Comparison of a target image with prediction images resulting from a model trained with 20 or 1280 images. The ML training set consisting of 20 images accurately predicts boundaries of cells but fails to identify small spindle-like subcellular structures (arrows). (**b**, **c**) Performance of fluorescence image predictions measured by image-wise Source and Laplacian Pearson correlation coefficients as a function of the number of training images. A higher number of training images improve the predictions of details and rare events. For both boxplots, ns stands for not significant; *p < 0.05; **p < 0.001; ***p < 0.0001. (**d**) Simulating weakly expressed markers with step-wise lowering illumination. The first column displays immunofluorescent target images and the second and third columns represent a magnified target and prediction image, respectively, with adjusted brightness for better visualization. (**e**, **f**) Prediction performance across different excitation levels. Dim markers can be represented fairly robustly by our AI algorithm, with prediction differences primarily visible for small scale features such as detailed protein localization and fine morphological shapes. Dashed line in (**e**) indicates the theoretical maximum correlation between the target and optimal prediction images ($C_{max}$). Error bars represent the standard deviation of the mean. All box and point plots were created by using OriginLab version 2019.

First, we analyzed differences in prediction accuracy by modulating the number of training images. We found that with only 20 training images, relatively accurate predictions can readily be achieved by the ML model (Fig. 2a). Nevertheless, more training images still improved the prediction outcome, particularly for the local intensity variation, as shown in the rightmost column of Fig. 2a. This observation is also illustrated by the analyses of $r_s$ (Fig. 2b) and $r_{lap}$ (Fig. 2c).

We also tested the sensitivity of our ML model to input images with varying signal-to-noise ratios (SNR). In doing this, we lowered the microscope illumination power stepwise and repeated the data acquisition and training process for nine different intensities (Fig. S6). This experiment aimed to simulate the imaging of fluorophores with different emission intensities. Within the SNR range explored, we found that our ML model is able to reliably reproduce the input images without overfitting (Fig. 2d). We also found that our model smoothed the intensity distribution in low SNR images and effectively denoised the data, consistent with previous findings^40,48^. To summarize the relationship between the prediction accuracy and image brightness, we plotted $r_s$ (Fig. 2e) and $r_{lap}$ (Fig. 2f) as a function of SNR. We also added a theoretical upper bound $C_{max}$ that assumes the pixel-level noise is uncorrelated for comparison (“Methods”). We found that $r_s (SNR)$ qualitatively followed the trend of $C_{max}(SNR)$, maintaining a high value $>0.8$ throughout all tested SNRs (Fig. 2e). However, $r_{lap}$ showed a more significant dependence on SNR, illustrated by its nearly halved value at SNR $=8$ (Fig. 2f). We further identified that this more pronounced decay is attributed to both the weakly-correlated noise fluctuation and the less-accurate prediction of local intensity (Fig. S7).

Lastly, we evaluated the impacts of the impurities in the images (e.g., microscope slide dusts, fluorescent speckles, non-specific binding) on the CNN training (Fig. S8). We tested three cases, in which either the training or test (for making new predictions) images contain impurities or both of them show artifacts. Our results suggested that the training set quality has a greater impact on the prediction accuracy than the test set quality (Fig. S8). This suggests that careful sample preparation and quality control of the training set plays a crucial role in achieving optimal prediction outcomes. In addition, we found that while impurities in the phase contrast images can propagate through to the prediction, most of the fluorescent speckles that only appear in the target images can be suppressed through ML training (Fig. S8a).

### Multi-marker heterogeneity

**Figure 3:**
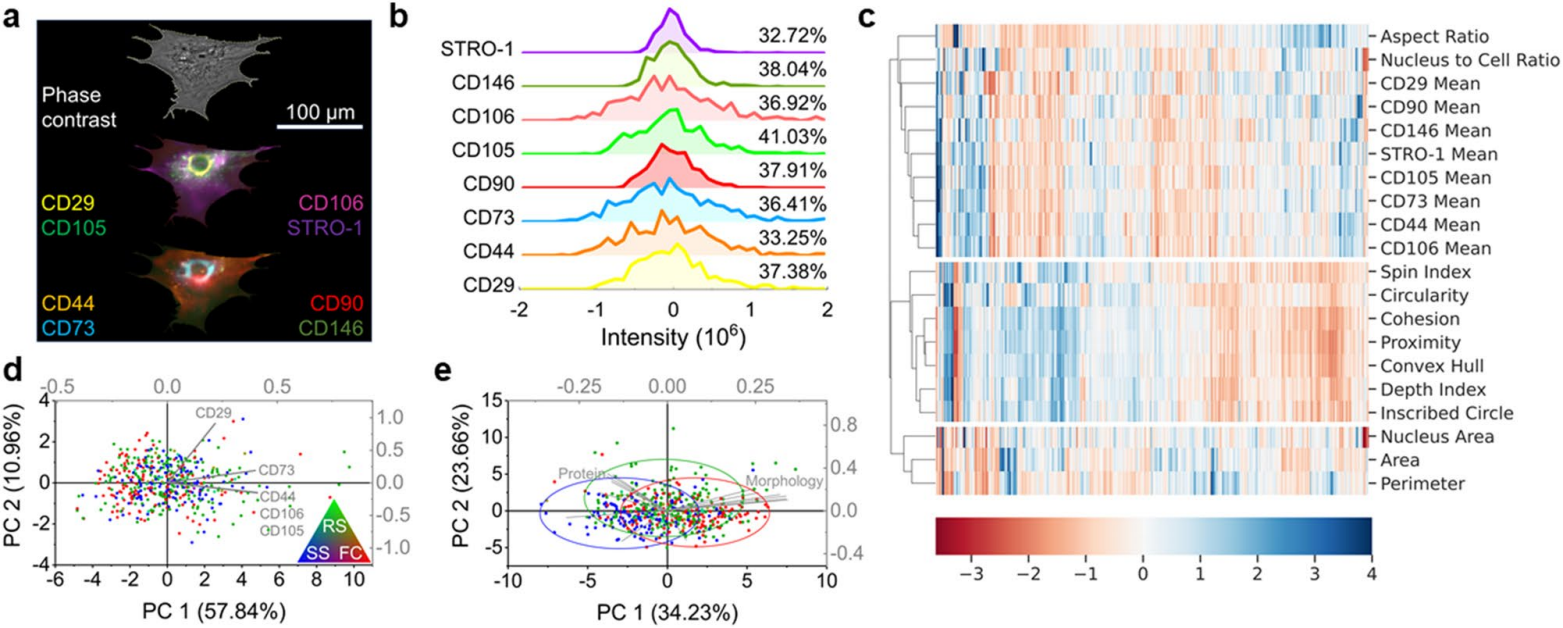
Multi-marker heterogeneity and gene expression-morphology correlation. (**a**) Example of cell outlining and marker distribution for single cell analysis. (**b**) Histogram of standardized intensity distribution of 500 single cells for 8 MSC markers. Percentages illustrate the standard deviation of each intensity distribution. Histogram was created by using OriginLab version 2019. (**c**) Hierarchical clustering of marker intensities and 12 different morphological features analyzed from outlined cells. Marker intensity and morphological features lead to distinct clustering patterns. The color represents the fold change with respect to the mean value for each row. Clustering analysis (heatmap) was performed using Python version 3.8 (seaborn). (**d**) Principal component analysis (PCA) biplot of mean intensity of all tested surface markers with loading vectors (grey) of the five most prevalent markers. (**e**) PCA of marker intensity and morphological features color-coded based on Haasters’s classification^54^. Green, blue, and red denote rapidly self-renewing (RS), spindle-shaped (SS), and flattened-cuboidal (FC) phenotyes, respectively. Relatively orthogonal loading vectors suggest low correlation between morphology and marker intensity. PCA was performed using OriginLab version 2019.

Our AI-based imaging approach opens up the possibility to perform a multi-marker characterization of MSCs in situ, which has been suggested to provide a more accurate description of the cell state^27,49^. Compared to multi-color fluorescence-activated cell sorting (FACS), which has been commonly used for similar subpopulation analyses, our method is free from spectral overlaps and does not require complex compensation procedures^50^. Specifically, since each model (marker) was trained with only one fluorescent channel at a time, the final multi-color composite is simply a combination of predictions from individually trained models. This training process is free from the influence of protein colocalization, and, theoretically, allows us to include unlimited markers for characterizing cells. We performed a single-cell analysis (Fig. 3a, Fig. S9) of 500 cells where we measured the overall fluorescent intensity over individual cells for all eight markers (Fig. 3b). This microscopy-based quantitative measurement has been extensively validated and conducted for evaluating gene expression^51–53^. After calculating the cell-level overall pixel intensity, we plotted the prediction value versus the target value and determined the corresponding correlation coefficient (Fig. S10). Consistent with the pixel-to-pixel comparison (Fig. 1d), we also observed high prediction-target correlations for all tested markers.

In addition to quantifying the gene expression level, our approach also allowed us to simultaneously characterize the MSC phenotypic morphology (Fig. S11, “Methods”), as it has been shown that non-invasive microscopy can potentially be used to predict the stem cell fate of human MSCs for clinical applications based on morphology^19,55^. Such integrative gene-morphology datasets then enabled us to directly elucidate the relationship between such essential cell properties. To visualize the correlation between the tested 20 variables (8 MSC markers and 12 morphological features), we performed an unsupervised hierarchical clustering analysis and created the resulting heatmap shown in Fig. 3c. The clustering indicated that while both MSC markers and cell morphology were able to separate cell subpopulations, their identified heterogeneities exhibited distinct patterns. Specifically, among all tested morphological features, we found that the cell aspect ratio and nucleus-to-cell ratio have higher correlations to MSC markers as they clustered together far from the rest of the morphological features. In contrast, nucleus area, cell total area, and perimeter show a clustering result that is less correlated with all other properties explored. Moreover, our data suggest that MSC markers provide additional orthogonal assessments on cell behavior that may not be fully revealed using the morphology analysis alone.

To further validate this finding, we conducted a Principal Component Analysis (PCA) to examine the correlation between MSC marker expression and cell morphology (Fig. 3d,e). In addition, we used a previous morphology-based categorization protocol^54^ to divide the cells into three subgroups: elongated and spindle-shaped cells (SS, blue); small, triangular or star-shaped cells (RS, green); and large, flattened cells with prominent nuclei (FC, red). This additional characterization data was incorporated into our PCA gene-morphology plot to validate previous findings. In the PCA biplot, where only the MSC markers were used (Fig. 3d), we did not observe any segregation of the data points. As expected, after including the morphological features in the PCA (Fig. 3e), these previously categorized subpopulations showed indications of separation. This incomplete segregation may be attributed to human errors in classifying the cells based on visual grouping.

Our observed low correlation between surface marker expression and morphology are consistent with previous studies^19,56–59^. For example, it has been shown that expression of MSC markers does not directly report the proliferation capacity and senescence of MSCs^56^. Also, recent experiments have used the morphological phenotype to better quantitatively predict the MSC immunomodulation potency^19,57^ and differentiation potential^58,59^. In fact, many MSC markers were originally designed to exclude hematopoietic cells that are usually co-harvested during isolation. While they can effectively validate the identity and origin of MSCs^27,60,61^, they may be insufficient to describe the cell state and function. Collectively, our gene-morphology data suggested that combining surface protein marker expression with morphological features could provide a more complete and specific evaluation of MSC heterogeneity. This finding highlights another important advantage of conducting cell assessment using our non-invasive AI-based imaging method.

### Realtime gene expression measurement

**Figure 4:**
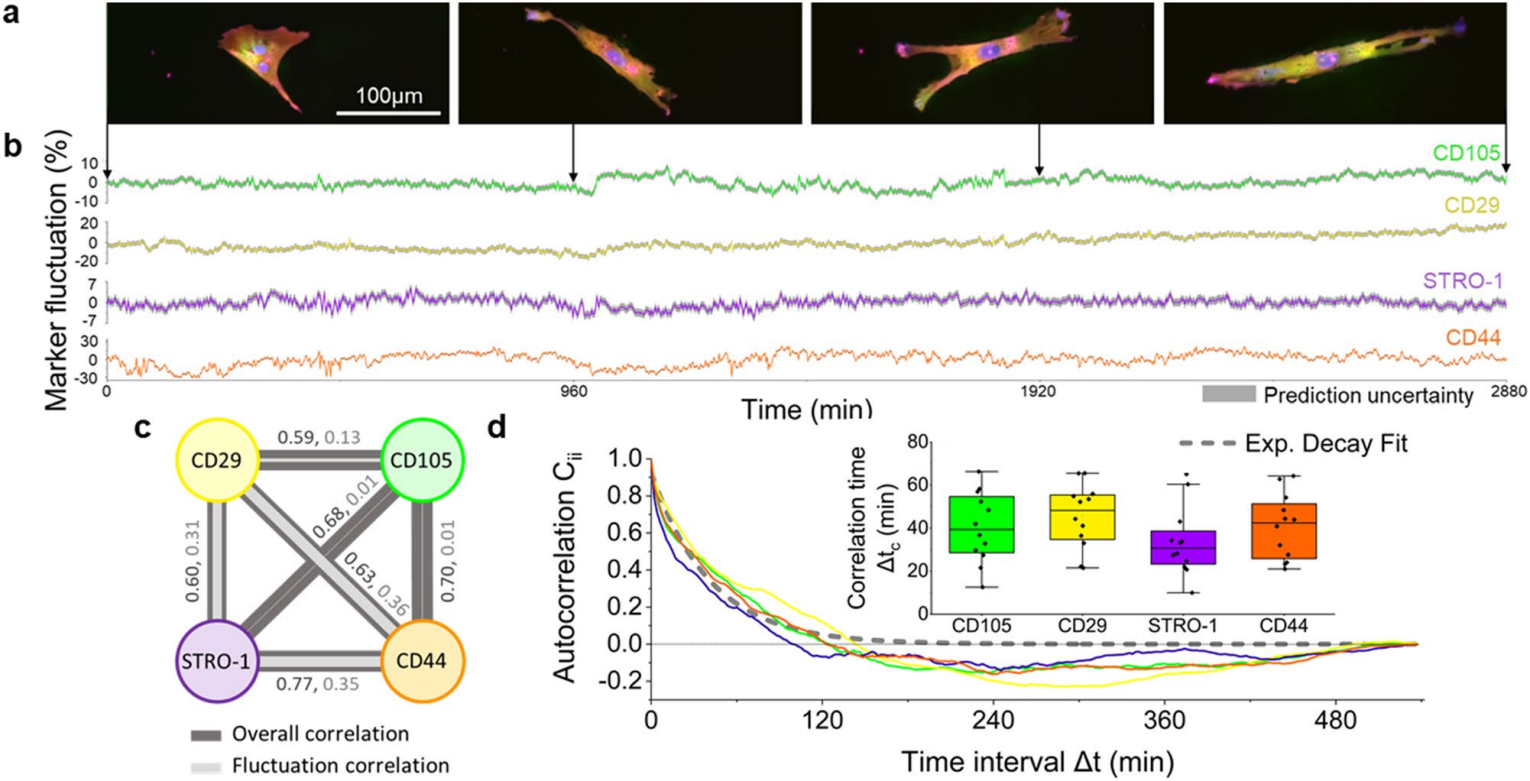
Spatial-temporal fluctuations of MSC marker expression. (**a**) Predicted time-lapse composite snapshots of a single cell at 0, 16, 32 and 48 h (left to right). (**b**) Total protein expression fluctuation for 4 surface markers (CD105, CD29, STRO-1, and CD44) of the predicted 48-h time-lapse video. Graph was created by using OriginLab version 2019. (**c**) Pairwise cross correlation between the tested markers. We calculated both the overall correlation (dark grey) and the fluctuation correlation (light grey) where the line thickness denotes the correlation magnitude. The fluctuation correlation (light grey) calculated using the single cell data is significantly lower than the overall correlation determined using all 500 cells, indicating remarkable stochasticity in the gene expression fluctuation. (**d**) Averaged autocorrelation functions (ACF) for all four tested markers exhibit a similar exponential decay. Inset shows the ACF decay rates, which are roughly the correlation time of them. (**d**) was created by using OriginLab version 2019.

Realtime gene expression assessment is crucial to evaluate MSC culture quality for basic research, therapeutics, and drug screening^12^. Our AI-based microscopy allows us to perform instantaneous gene expression measurements on both the cellular and subcellular levels. Demonstrating this technique, we acquired a phase-contrast time-lapse video of live MSCs over 48 hours and generated the corresponding AI-predicted fluorescent videos for 4 MSC markers, CD105, CD29, CD44, and STRO-1 (Fig. 4a, Videos S1, S2). We validated the usage of ML models of fixed samples for predicting live cells by testing the influence of the fixative on the cell morphology. We found that no significant morphological change could be observed (Fig. S12). As shown in the predicted video, we observed significant fluctuations in the gene expression level, reminiscent of the stochastic gene expression findings in other systems^62,63^. We analyzed and plotted the evolution of the overall expression level of a single cell in Fig. 4b. To validate the observed fluctuation, we highlighted the 60% confidence interval of the predicted value with gray bands (Fig. 4b), which was estimated by analyzing the distribution statistics of the prediction-target error, see Fig. S13. This prediction uncertainty was found to be $<2$% for all markers (Fig. S13c).

To understand the relationships of the fluctuations between MSC markers, we analyzed two types of marker cross-correlations. The first analysis averaged the overall cross-correlation values over different cells for a single time point. This calculation was performed using the dataset shown in Fig. 3. The second analysis, in contrast, averaged the marker cross-correlation over the entire temporal fluctuation for a single cell (Fig. 4b). By comparing these analyses (Fig. 4c), we found that the fluctuation correlation (averaged over time) is significantly lower than the overall correlation (averaged over cells) for all tested markers. This reduced correlation value indicated that the observed temporal fluctuations are strongly influenced by uncorrelated noise, consistent with previous stochastic gene expression experiments^64–66^.

While these fluctuations are relatively independent, we found that all markers exhibited a similar autocorrelation decay trend (Fig. 4d). Here, the autocorrelation function characterizes the persistency of a temporal fluctuation. By fitting an exponential decay to the head of the curve, we found that all four markers displayed a correlation time around 40 minutes (inset of Fig. 4d). The minor anticorrelation in Fig. 4d may be attributed to the finite measurement duration^50^.

**Figure 5:**
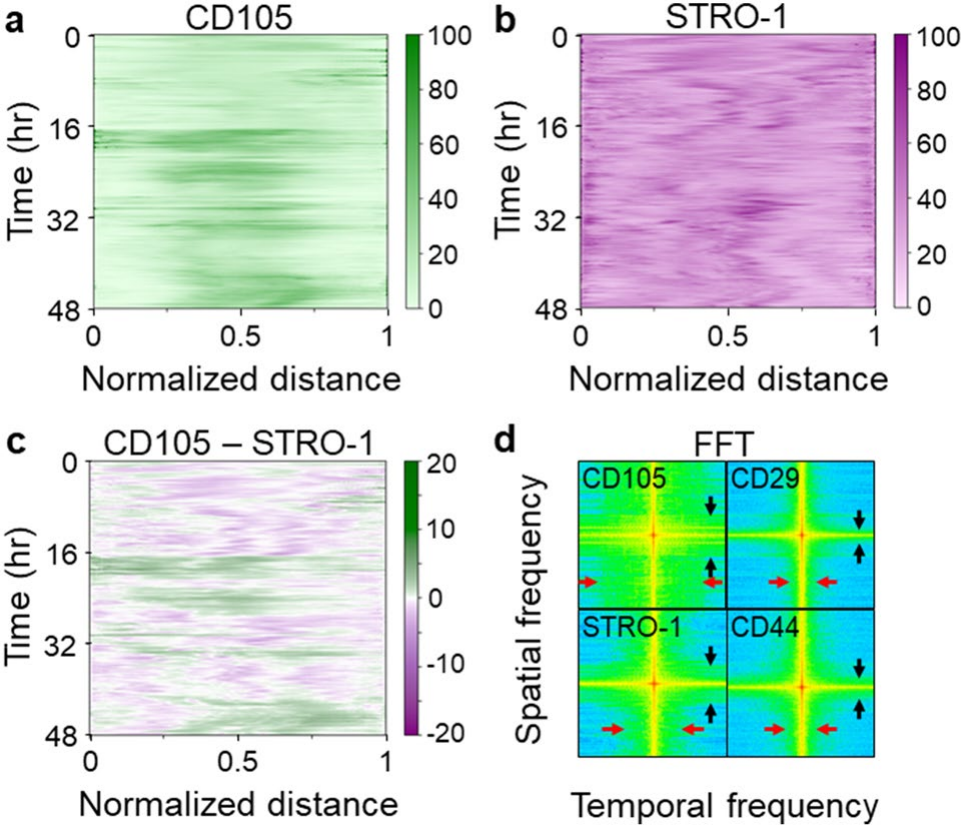
Prediction of temporal marker expression holds information on intracellular heterogeneity. (**a**, **b**) Heatmaps showing intracellular marker distribution and fluctuation for typical MSC surface markers. Fluctuation patterns vary significantly between tested markers indicating strong marker dependency within a single cell. (**c**) Protein localization differences of CD105 and STRO-1 underlining marker dependency. (**d**) 2D Fast Fourier Transform of heatmaps (**a**, **b**) revealing oscillatory frequency differences in time and space. Red and black arrows highlighting characteristic temporal and spatial fluctuation lengths, respectively. CD105 shows highest temporal and spatial fluctuation. Subfigures were generated using the OriginLab software version 2019 (**a**–**c**) and Fiji ImageJ^79^ software (**d**).

Lastly, we analyzed the intracellular heterogeneity using the acquired multi-marker time-lapse data (Video S2). We calculated a projected intracellular heterogeneity by averaging the gene expression field over the vertical axis and determined the 1-dimensional profiles of gene expression level across the cell (Fig. S14). This calculation was then repeated for each time point, in which the final spatial-temporal matrices are illustrated using heatmaps shown in Fig. 5a–c. The heatmaps illustrated clear subcellular differential expression between all markers (CD105, CD29, STRO-1, CD44) throughout the cell and over time. CD105 (Fig. 5a) and STRO-1 (Fig. 5b) showed the strongest expression difference. This differential expression was nearly 50% of the analyzed gene expression fluctuations (Fig. 5c). The distinct fluctuation frequency and persistency of these protein distributions were shown by the 2D Fast Fourier Transform (FFT) plots (Fig. 5d), in which CD105 displayed a temporal (red arrows) and spatial (black arrows) fluctuation that is more significant than that for STRO-1, CD29, and CD44. Our findings collectively indicated that the characteristics of the intracellular heterogeneity, which can be evaluated by the protein localization, are strongly gene dependent. While it would be interesting to further validate these findings using fluorescent reporters, creating such a multi-marker reporter line is challenging and beyond the scope of this work. Nevertheless, all of our analyses mainly focused on the statistical characteristics of the spatial-temporal gene expression fluctuation (e.g., correlation time and 2D FFT), minimizing the impact of potential ML prediction errors on the findings.

## Discussion

Overall, we demonstrated that AI-based label-free microscopy offers a powerful experimental platform for conducting non-invasive, quantitative, and multi-marker characterizations of MSC heterogeneity. With this tool, we found that the MSC morphology and conventional surface markers provide complementary assessments of the cell characteristics. On the cellular level, we showed that the stochastic gene expression fluctuations for all tested markers shared a similar correlation timescale. On the subcellular level, we observed and quantified the spatial-temporal variation of surface protein distribution. As a proof of concept, this work only focused on the MSC heterogeneity within a single-donor culture for similar passage numbers and seeding density. In the future, it would be informative to utilize a similar approach to investigate the heterogeneity associated with cell passage number, senescent state^67^, donor variation, and media composition, factors that have been shown to have significant impacts on MSC marker expression. Specifically, studying the relationship between MSC heterogeneity and replicative senescence would be particularly interesting, as it has been shown to be profoundly related to the characteristic, proliferation capacity, and multilineage potency of MSCs^68–70^. Furthermore, in addition to the tested classical MSC markers, several genes (e.g. indolamine-2,3-dioxygenase (IDO)^71^, alkaline phosphatase (ALP) and CD271^72^) have been recently shown to relate closely with MSC functions, providing an interesting opportunity for future study.

While AI-based microscopy presents many advantages over standard immunochemistry assays, many challenges persist. For example, it remains unknown whether the distribution of highly localized proteins (e.g. vesicle structures) or RNAs (e.g, telomeres), which are strictly invisible in the phase contrast images, can be accurately predicted by the ML models. Also, the black-box nature of CNNs provides limited information on how the model extracts transmitted light image features and translates them into fluorescent signals^31,73^. Furthermore, to apply this approach to high-throughput screening, it is crucial to develop reliable and automatic cell segmentation tools^74^.

Nevertheless, the current imaging platform can already enable many exciting applications in both basic and translational studies of MSCs. For instance, by studying the gene expression fluctuation during cell division, it is possible to address the clonal heterogeneity origin, which has been hypothesised to be associated with asymmetric partitioning of MSCs^75^. By introducing relevant receptor, pathway signaling, and oxidative stress genes, our method will also advance the specificity, readout dynamics, and throughput of toxicity and pharmacokinetic studies. Broadly, the ability to instantly quantify MSC characteristics paves the way for controlling cell source quality, optimizing culture conditions, and engineering specific cell functions, all critical steps for advancing current MSC-based cell therapies.

## Methods

### MSC culture

Human bone marrow-derived MSCs (ATCC, PCS-500-012) were cultured according to the manufactures instruction and previously published protocols^76,77^. In brief, after the MSCs were thawed, they were seeded into tissue culture flasks at a density of 5000 cells/cm$^{2}$ with the culture media comprising DMEM (Gibco, 1 g/mL glucose, 500 mL), 10% fetal bovine serum (Gibco), and 1% Penicillin/ Streptomycin (Gibco). The MSC culture media was then replaced every 24–48 h. Subculture of MSCs was performed at $\sim 80$ % confluency, in which the cells were washed with 1$\times$ PBS −/− twice, and incubated with 0.5% Trypsin-EDTA at 37 $^\circ$C for cell detachment. The dissociated cells were then centrifuged at 250*g* for 3 min, resuspended in warmed culture media and reseeded at a seeding density of 5000 cells/cm$^{2}$. For experiments, we seeded passage-3 MSCs into 2-well Ibidi slides (Ibidi, 80296) at a seeding density of 10,000 cells/cm$^{2}$. The experiment was carried out at least 24 h after seeding to ensure cell attachment.

### Sample preparation and immunostaining

To fix and immunostain the MSC samples, each Ibidi slide was first washed with PBS +/+. 4% PFA (Thermo Fisher Scientific, 28908) in 1$\times$ PBS +/+ (Gibco) was used as the fixative. After 5–10 min of incubation, the slides were washed with PBS. For staining, the MSCs were first blocked using a mixture of 2% donkey serum (Sigma-Aldrich, D9663-10ML) and 0.5% Triton X-100 (Sigma-Aldrich, T8787-50ML) for 30 min. After blocking, the slides were washed with PBS twice, and then incubated with the primary staining solution (0.5% BSA, 0.25% Triton X-100, and the primary antibody, see Supplementary Table S1). The slides were left in the staining solution for 30 min and then washed twice with 1$\times$ PBS. Afterwards, the secondary staining solution (with NucBlue and the secondary antibody) was added for 30 min. We then washed the samples twice with PBS and added 0.1% Tween 20 (Sigma-Aldrich, P9416-50ML) for storage.

### Transmitted light microscopy and fluorescent imaging

All stained MSC samples were imaged using an inverted microscope (Etaluma LS720, Lumaview 720/600-Series software) with a 20$\times$ phase contrast objective (Olympus, LCACHN 20XIPC) that allowed the acquisition of both phase contrast and fluorescence images. Approximately 600–900 images for each channel (i.e., phase contrast, 405 nm, 488 nm, and 594 nm) were obtained with a field of view $\sim 380\;\upmu$m $\times 380\;\upmu$m. To conduct the time-lapse experiment, the same microscope and software was used in an incubator to image MSCs every 2 min over a period of 48 h (Videos S1, S2). While all of our selected antibodies have been previously validated, we also examined the non-specific binding by measuring the fluorescent intensity in samples that were only stained with secondary antibodies. Prior to further analyses, the background of the fluorescent data was evaluated and subtracted, following typical quantitative immunofluorescent microscopy procedures.

### CNN model training and characterization

Our model, consisting of a pair of generator and discriminator, was adapted from a previous image-to-image translation work^40^. Specifically, we constructed our machine learning code using a Python deep learning library, PyTorch, ensuring that our AI platform is compatible with different operating systems. The architecture of the generator was the standard U-Net that has been widely used for image segmentation. In this work, it was modified as an image translator that transformed the phase contrast images into fluorescent-like data. In particular, the used U-Net model contained short-cut connections between hidden features at the same level, effectively combining the fine-grained input details with the high-level semantics of shapes. The adapted discriminator was a multilayer CNN that used the concatenation of signal and target images as input, and generated a tensor containing the information for judging the prediction-target similarity. Instead of adopting the typical approaches to solving the classification problem in GAN, we utilized the least square loss, which has been shown to be a more stable function^78^. Different CNNs were trained to predict the fluorescent images for individual markers, with the training data consisting of approximately 600–1500 pairs of phase contrast (input) and immunofluorescent (target) images.

To quantify the prediction accuracy of the CNN, the Pearson correlation coefficient between the target *x* and prediction *y* pixel intensities was evaluated as $\sum _{i=1}^{n}(x_i-{\bar{x}})(y_i-{\bar{y}})/ \left[ \sum _{i=1}^{n}(x_i-{\bar{x}})^2\sum _{i=1}^{n}(y_i-{\bar{y}})^2 \right] ^{1/2}$. This calculation was applied to both the images and their corresponding Laplacian fields (kernel size = 31) in Python to obtain $r_s$ and $r_{lap}$, respectively (Figs. 1d,e, 2b,c,e,f). The $C_{max}$ curve shown in Fig. 2e was evaluated using $C_{\max }=\frac{{\mathbb {E}}[Y\cdot {\hat{Y}}]}{\sqrt{{\mathbb {E}}[Y^2]{\mathbb {E}}[{\hat{Y}}^2]}}=\sqrt{\frac{SNR}{1+SNR}}$ with ${\text {SNR}}={\sigma ^2_x}/{\sigma ^2_{\epsilon }}$ , which determined the upper bound of the target-prediction correlation where the signal noise is uncorrelated. To calculate the SNR values of the fluorescent images used for Fig. 2d–f, we used the Root Mean Square (RMS) method with the following formula: ${\text {SNR}}={(S_{signal}-S_{noise})}/{N_{noise}}$. The mean ($S_{signal}$ and $S_{noise}$) and standard deviation ($N_{noise}$) of pixel intensities were measured using Fiji ImageJ^79^.

### Single cell measurement

We used the Fiji ImageJ^79^ polygon selection tool to manually outline 100 cells for both the immunofluorescence images and the corresponding ML-predicted images. The outlined cells were then analyzed to evaluate the overall pixel intensity and the prediction accuracy (Fig. S9). We then repeated this outlining procedure to obtain 500 cell data points, in which the data were used for the multi-marker analysis and morphology measurements (Fig. 3). PCA (Fig. 3d,e) was performed using OriginLab (OriginPro, Version 2019. OriginLab Corporation, Northampton, MA, USA). The unsupervised clustering analysis (Fig. 3c) was performed using Python 3.8 (seaborn). Figures 3b and 4b,d were generated using OriginLab where for Fig. 4d, the autocorrelation function of Excel (RealStatistics package) was used. The heatmaps displayed in Fig. 5a–c, were obtained using Python v3.8 described in the Supplementary Information (Fig. S14). We used the Fiji ImageJ^79^ FFT command to compute the Fourier transform and display the frequency spectrum of the heatmaps displaying intracellular marker distribution. Heatmaps were converted to gray scale prior to FFT computation and a density-based 16 color look up table was applied to the resulting FFT to highlight the spectrum pattern (Fig. 5d).

## Supplementary information

Supplementary Information.

Supplementary Video S1.

Supplementary Video S2.

## Data Availability

*Accession codes* Software for training and an example dataset is available https://xuanqing94.github.io/ai-reporter/.
