# Supplementary material for: Investigating heterogeneities of live mesenchymal stromal cells using AI-based label-free imaging
Source: Sci Rep. 2021 Mar 24;11:6728. doi: 10.1038/s41598-021-85905-z (PMC7991643; doi:10.1038/s41598-021-85905-z)
Supplement: Supplementary file 1 — Supplementary Information. [file 41598_2021_85905_MOESM1_ESM.pdf]

Supplementary Information for

Investigating Heterogeneities of Live Mesenchymal Stromal Cells Using AI-based Label-free Imaging

Sara Imboden[1,†,*], Xuanqing Liu[2,†], Brandon S. Lee[3], Marie C. Payne[1], Cho-Jui Hsieh[2], and Neil Y.C. Lin[1,3,4]

This pdf file includes:
Figs. S1 through S14
Table S1
Captions for Movie S1 and S2
SI References

Other supplementary materials for this manuscript include the following:
Video S1 and S2

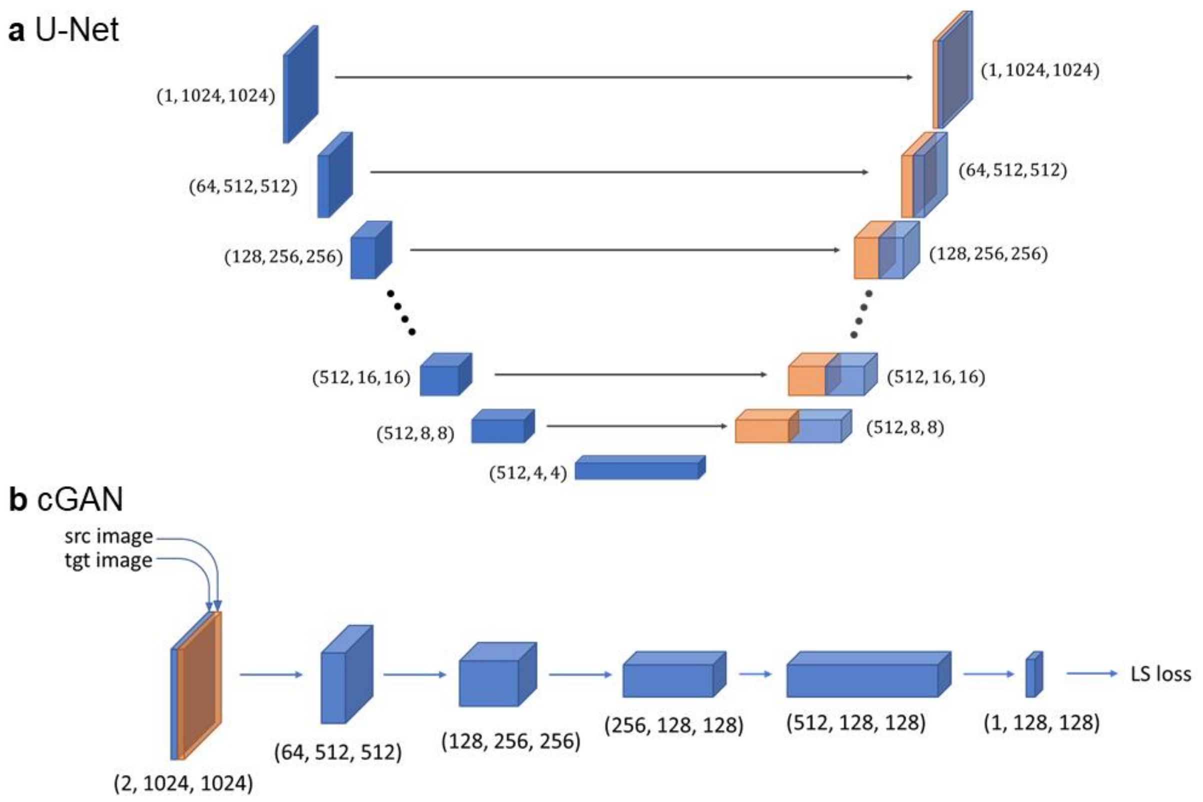

Figure S1. CNN architecture. a. The neural network architecture with sizes of tensors [39]. The utilized U-Net network served as the generator that converted a source image to a prediction. Blue boxes correspond to a multi-channel feature map and orange boxes represent copied feature maps. **b.** The cGAN neural network architecture with sizes of tensors. We used a plain multilayer convolutional neural network without shortcut connections [40, 80]. The number of channels is denoted in the bracket below each box.

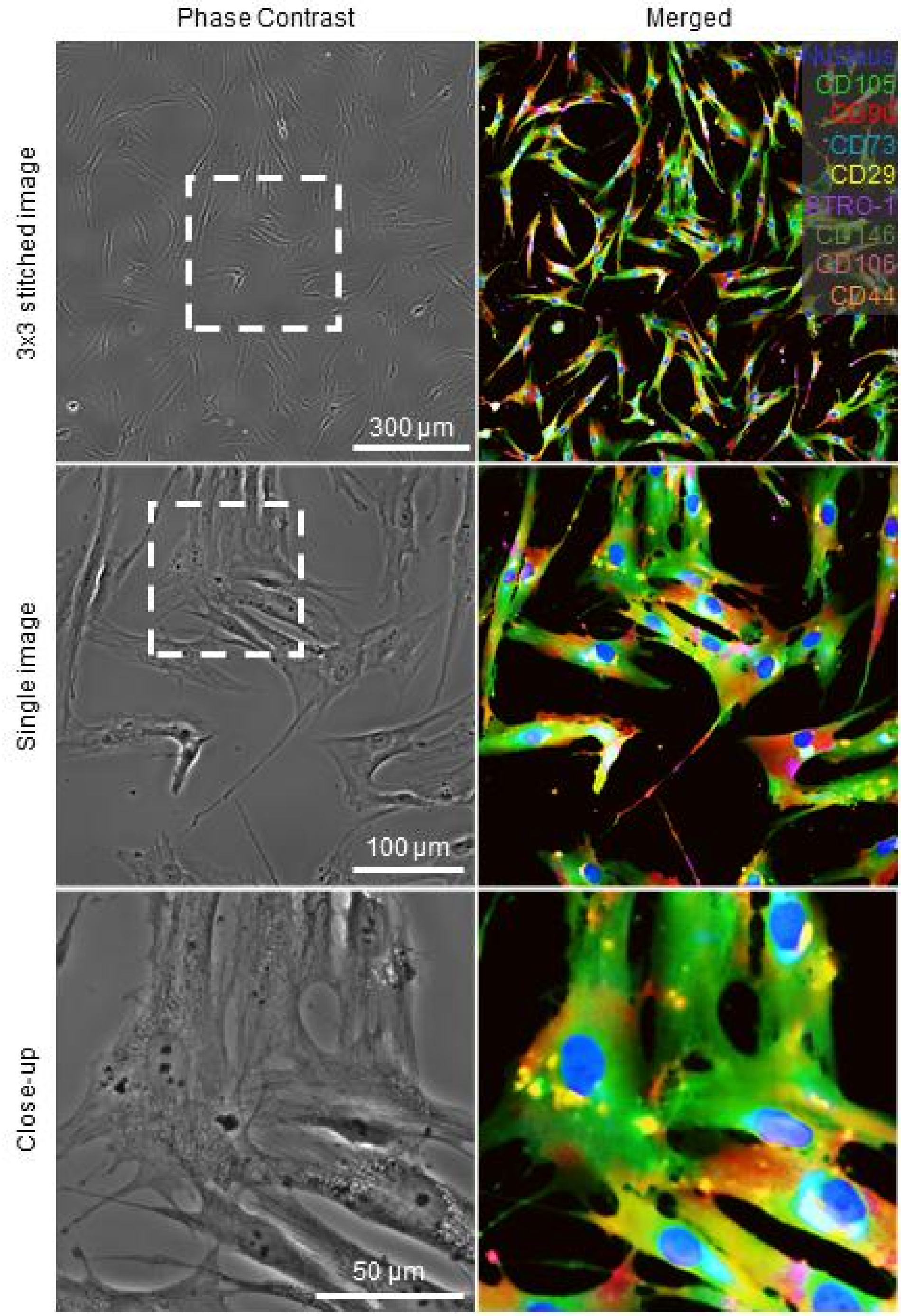

Figure S2. ML-predicted multiscale images. Multi-marker fluorescent images predicted from the phase contrast data. Left column shows the phase contrast images (input) and right column are the corresponding ML predicted composite images (prediction). Composite images were obtained by collapsing a stack of 9-marker images (Nucleus, CD105, CD90, CD73, CD29, STRO-1, CD146, CD106 and CD44). We show a 3x3 stitched image of MSCs (top), a zoomed-in single field of view (1024x1024 pixels, middle), and a close-up of a local area (bottom). The white dashed boxes denote the selected and zoomed areas.

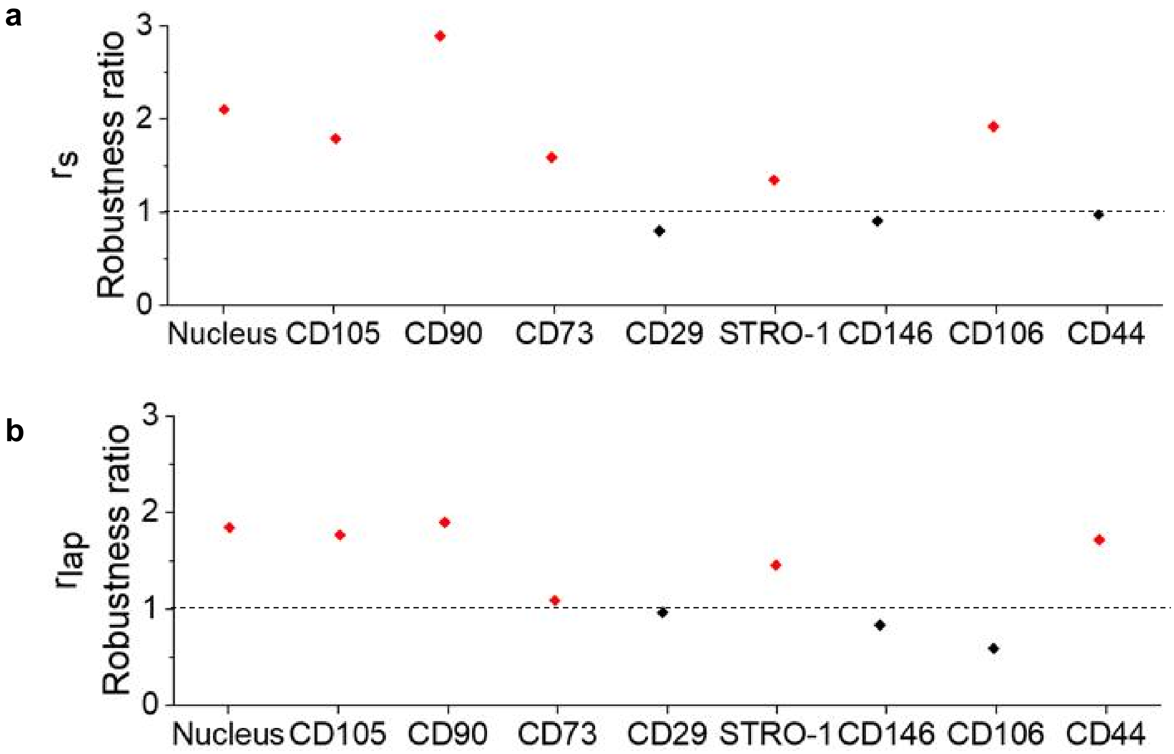

Figure S3. Prediction robustness comparison. We defined the robustness as the error bar size (1.5X interquartile) shown in Figs. 1d and e. The robustness ratio was then determined by dividing the U-net only value by the U-Net + cGAN value. For example, a robustness ratio > 1 suggested a more robust prediction from the U-Net + cGAN model. We found that the U-Net + cGAN model showed higher prediction robustness for 6 tested markers for both r_s(a) and r_{lap}(b).

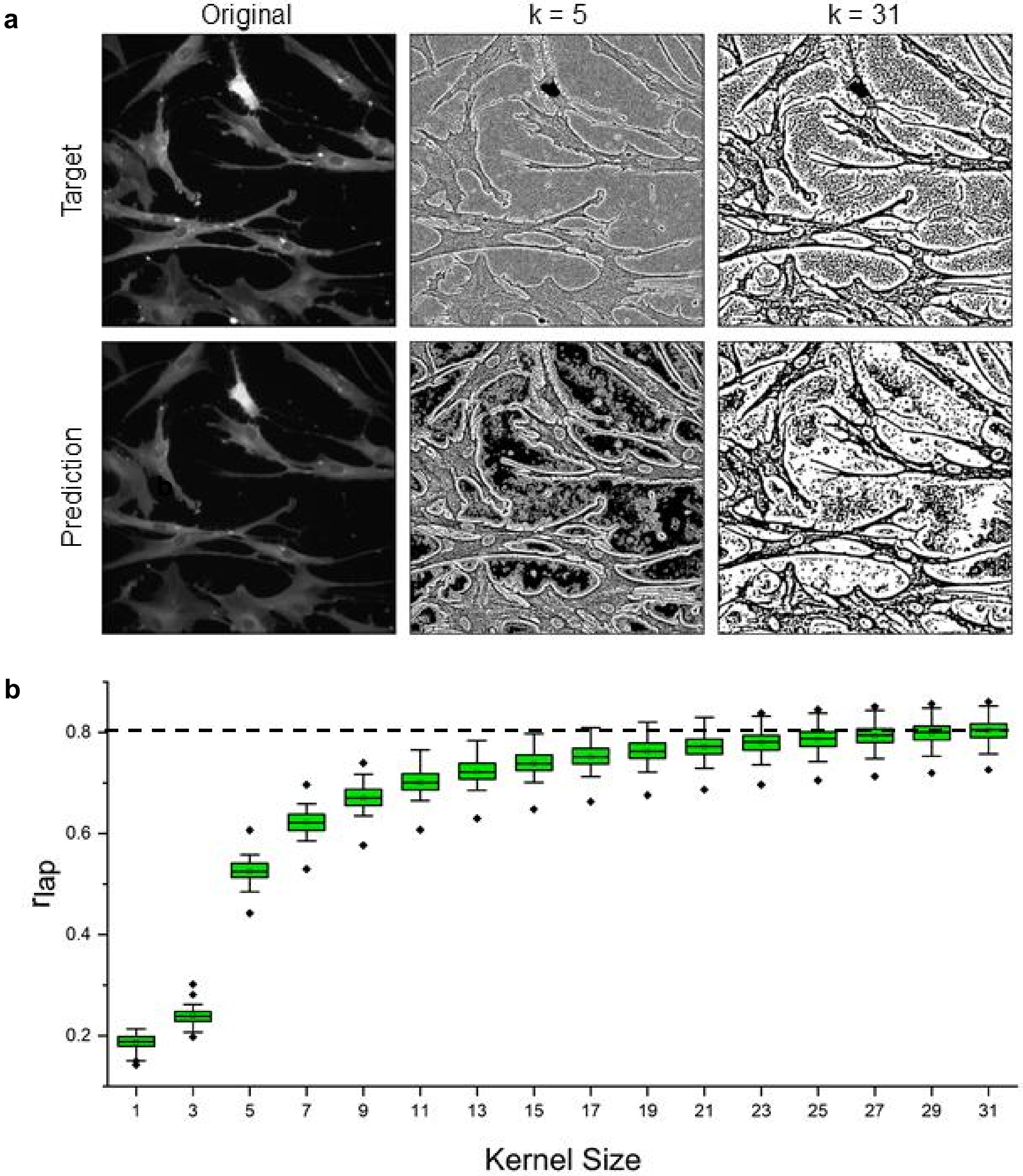

Figure S4. Pearson correlation coefficient of Laplacian. a. Image array displaying target and prediction of the original images (left column) and the corresponding Laplacian fields with kernel size k=5 (middle) and k=31 (right). Morphological details are clearly visible even with the largest kernel size tested in this analysis. **b.** Pearson correlation coefficient r_{lap} plotted as a function of kernel size k. We found that the value of r_{lap} became insensitive to the kernel size roughly at k ~ 27, indicated by the plateau. Based on this result, we selected k=31 as the kernel size throughout our quantitative r_{lap} measurements.

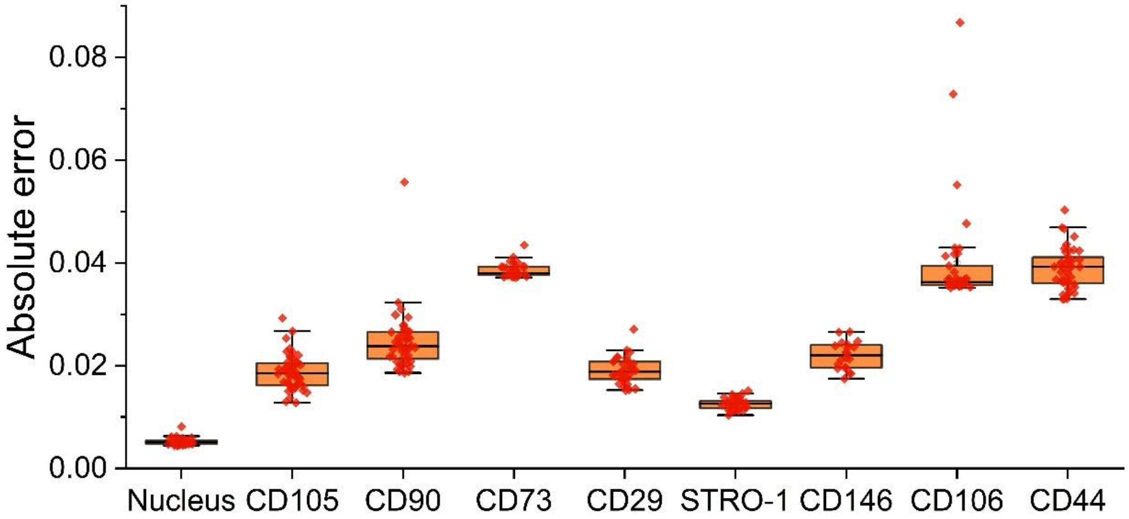

Figure S5. Prediction accuracy quantification: absolute error. In addition to the Pearson correlation coefficient, we computed the absolute error of the prediction accuracy. Absolute error was calculated between normalized pixel values (normalized by 255) of the target (ground truth) and prediction images. Retrieved results indicated an overall low mean absolute error for all markers, verifying the r_s and r_{lap} values shown in Fig. 1c and d.

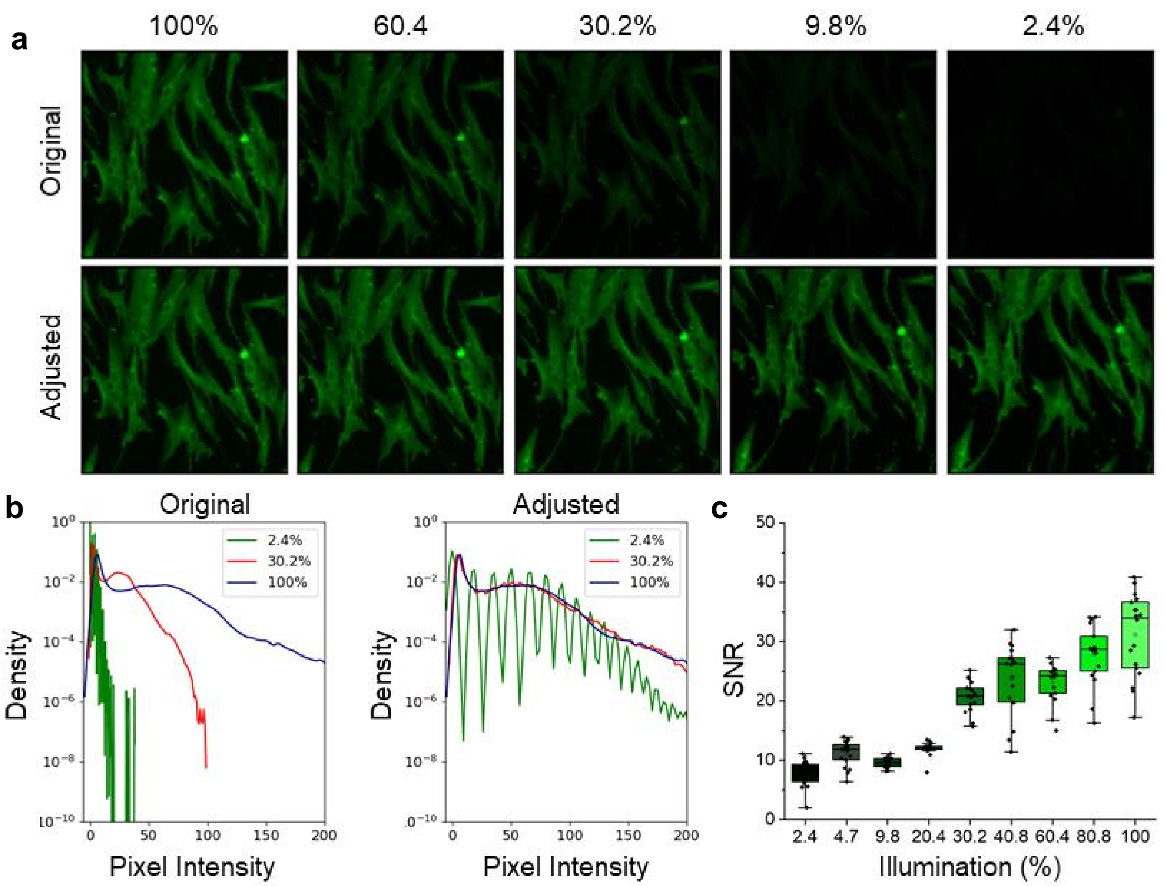

Figure S6. Stepwise illumination test. a. Image array showing example data for 5 excitation intensities. 11 identical field of views were utilized for direct quantitative comparison across different excitation levels. ML model accurately predicted overall fluorescent signal distribution (cell morphology) for all tested conditions. But the overall image quality decreased with decreasing signal-to-noise ratio (SNR). **b.** For better visual comparison, we adjusted the image brightness (Fig. S6a, bottom row) retroactively by standardizing the pixel value histogram. The numbers (2.4% green, 30.2% red, and 100% blue) correspond to the relative microscope illumination intensity. **c.** Boxplot showing that the SNR value monotonically increases with increasing Illumination intensity.

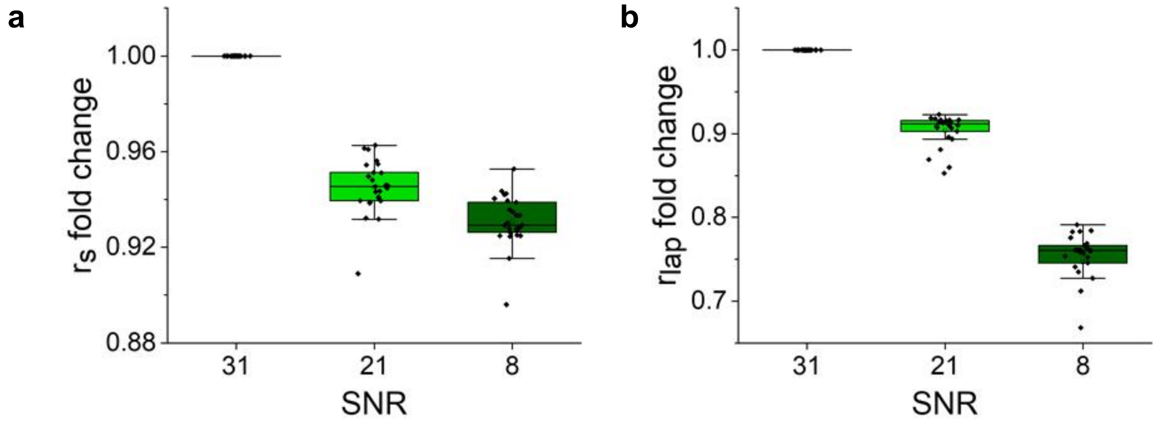

Figure S7. Prediction accuracy of dark images. We calculated the prediction accuracy (Pearson correlation coefficient) of images with 3 different SNR values, in which we averaged over 25 images**.** All data were normalized by the value at SNR=31. **a.** r_s value slightly decreased by <8% while SNR was reduced by 75%. **b.** In contrast, r_{lap} value exhibited a noticeable ~30% drop as SNR reduced.

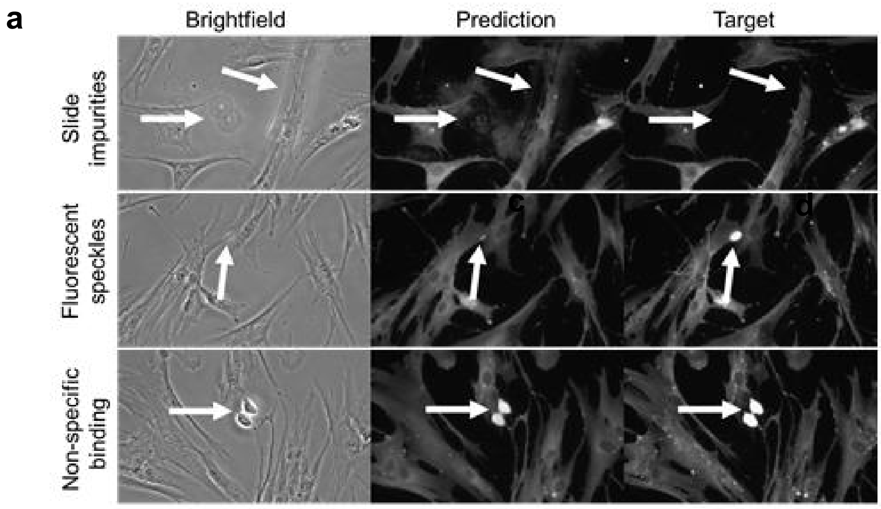

b

	Brightfield	Target	Prediction
Slide Impurities	✓	×	✓
Fluorescent speckles	×	✓	×
Non-specific binding	✓	✓	✓

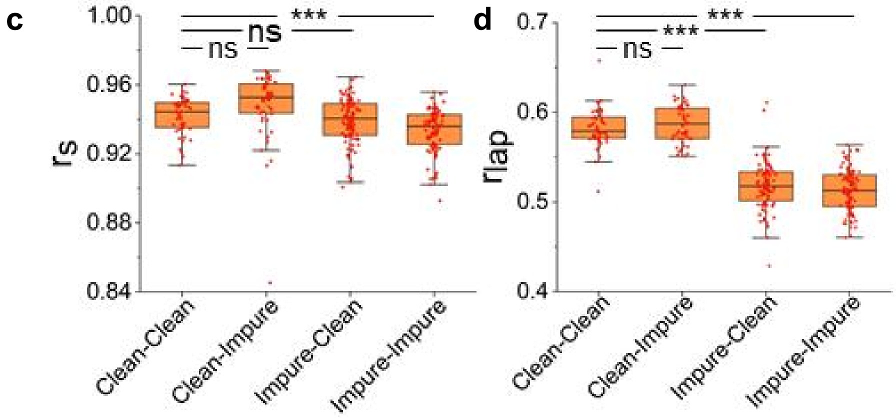

Figure S8. Image impurities influence ML predictions. a. We tested three types of image impurities: slide impurities (e.g. scratches, dust, bubbles) (top), fluorescent speckles (middle), non-specific binding of the antibody (bottom). The input phase contrast (left column), prediction (middle column), and target (right column) images are shown. Overall, if impurities are present in phase contrast image, it will be propagated in the prediction image (top and bottom rows). However, the artifacts that are only shown in the target fluorescent images can mostly be suppressed through model prediction (middle row). **b.** Table summarizing the qualitative observations made in Fig. S8a. The symbols ✓ and × indicate the presence and absence of artifacts in the image, respectively. **c-d.** r_s (c) and r_{lap} (d) values for four different training and testing conditions (the labels follow the format: training-testing). Here, the impure dataset consisted of 25% images that contain artifacts. Roughly 25% r_{lap} reduction was observed when impure dataset was used for ML training, implying the importance of image quality control.

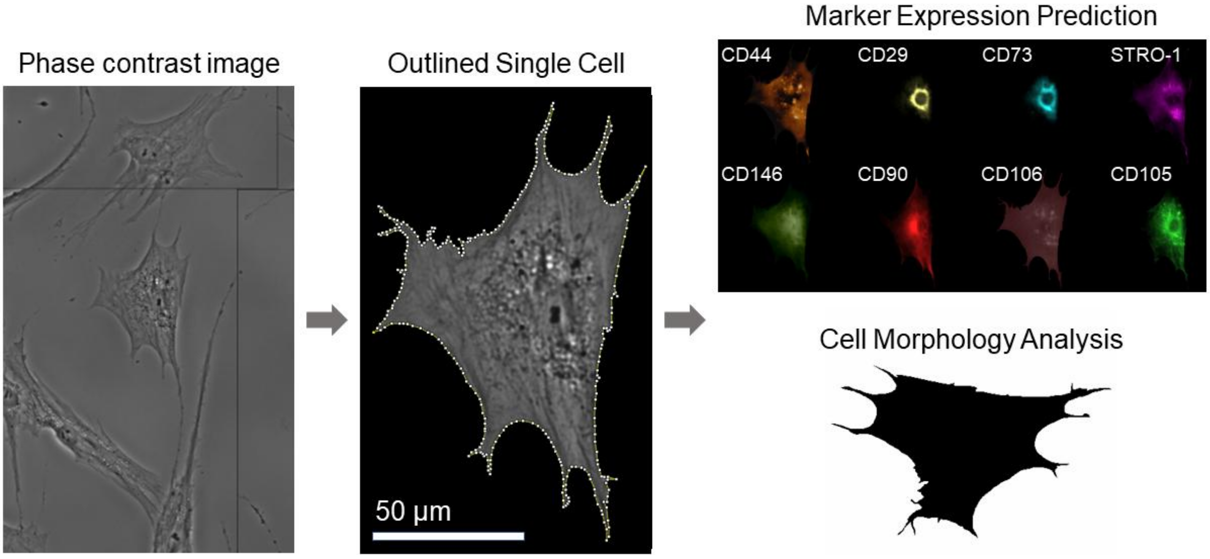

Figure S9. Cell Outlining Workflow. We obtained 10x10 tiling phase contrast images of a MSC sample and predicted immunofluorescence images for all studied markers (Methods). Using ImageJ, we manually outlined 500 cells (ImageJ polygonal selection tool) within the stitched composite images (phase contrast and fluorescent images) and created single cell image files. The left image shows a local field of view of the phase contrast channel in a stitched composite. The middle image shows an example of selected cell contour, closely capturing the detailed cell morphology. The selected area was than applied to all the predicted fluorescent channel (upper right image array) for further quantitative measurements. A combination of custom code (Python) and ImageJ was used for quantifying the pixel intensity measurements and morphology analysis (right bottom) for all 500 cells. The analyzed morphology features are summarized in Fig. S11.

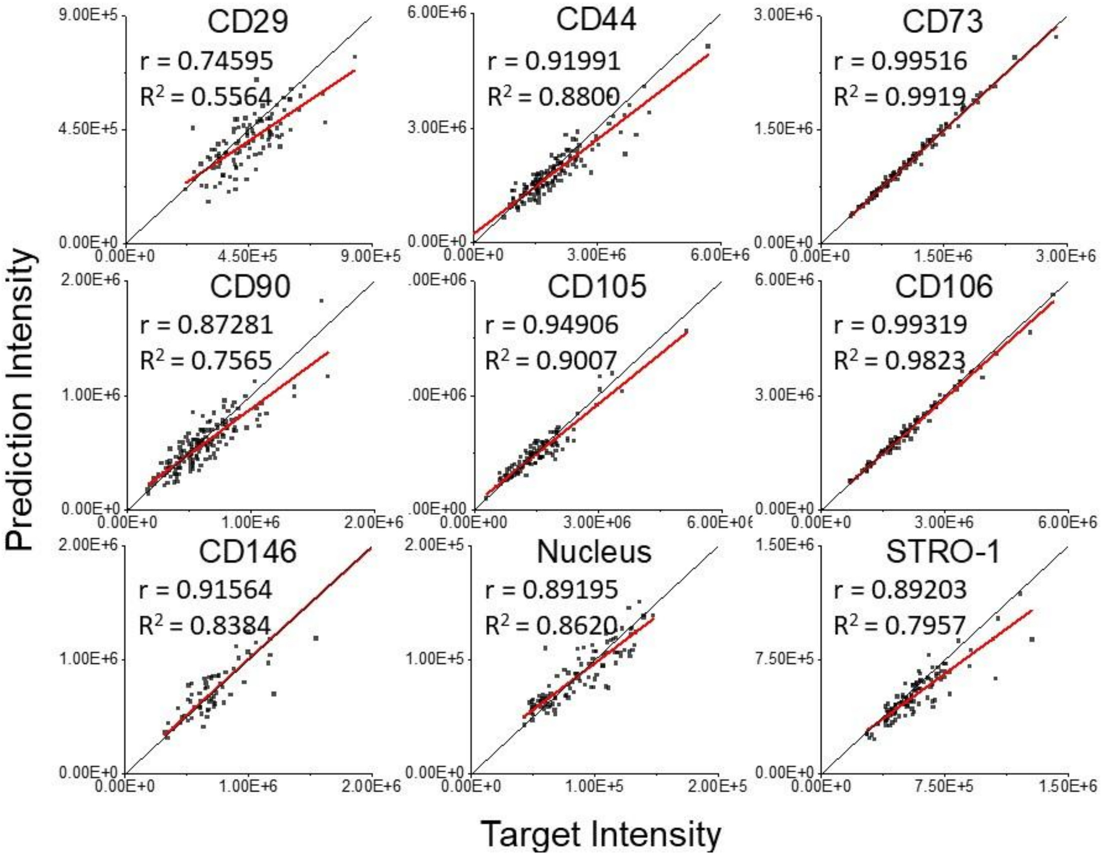

Figure S10. Single Cell Comparison. The sum of the pixel intensities of both single cell target images (immunofluorescence) and single cell prediction images were acquired using ImageJ (Fig. S9). The scatterplots demonstrate the comparison of each cell between the target intensity and the model's prediction of that fluorescent label (n = 100). The red and black lines correspond to the linear regression fit and x = y, respectively. The Pearson correlation coefficient r and coefficient of determination R^2 were calculated for each of these scatter plots and were displayed in the top left corner of each scatterplot.

Shape	Name	Description	Formula	Unit
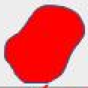	Area of the Cell	Area of the selection	Area of the Cell = Number of pixels contained within the selection * Pixel to Micrometer Conversion Factor for Image	μm^2
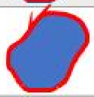	Perimeter of the Cell	Perimeter of the selection	Perimeter of the Cell = Number of pixels on the border of the selection * Pixel to Micrometer Conversion Factor for Image	µm
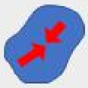	Proximity	The average Euclidean distance from all interior points of the selection to the centroid of the selection normalized to the equal area circle (EAC)*	Proximity = $Proximity_{EAC}$ / $Proximity_{Selection}$<br>$Proximity_{Selection} = (d_1+d_2+...+d_n)/n$<br>$Proximity_{EAC}$ = 2/3 * $Radius_{EAC}$**<br>[d = Distance from point to centroid, n = Number of points]	NA
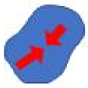	Spin Index	The average of the square of the Euclidean distances between all interior points of the selection to the centroid of the selection normalized to the EAC	Spin = $Spin_{EAC}$ / $Spin_{Selection}$<br>$Spin_{Selection} = (d_1^2+d_2^2+...+d_n^2)/n$<br>$Spin_{EAC}$ = 1/2 * $Radius_{EAC}$**2<br>[d = Distance from point to centroid, n = Number of points]	NA
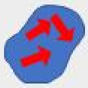	Cohesion	The average distance between all pairs of interior points of the selection normalized to the EAC	Cohesion = $Cohesion_{EAC}$ / $Cohesion_{Selection}$<br>$Cohesion_{Selection} = (d_1+d_2+...+d_n)/n$<br>$Cohesion_{EAC}$ = 0.9054 * $Radius_{EAC}$**<br>[d = Distance from point to point, n = Number of point pairs]	NA
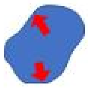	Depth Index	The average distance from the interior points of the selection to the nearest point on the perimeter of the selection normalized to the EAC	Depth = $Depth_{Selection}$ / $Depth_{EAC}$<br>$Depth_{Selection} = (d_1+d_2+...+d_n)/n$<br>$Depth_{EAC}$ = 1/3 * $Radius_{EAC}$**<br>[d = Distance from point to nearest point on the perimeter, n = Number of points]	NA
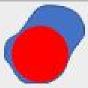	Inscribed Circle	The radius of the largest circle that can be inscribed in the selection normalized to the EAC	Inscribed Circle = $Radius_{Inscribed\ Circle}$ / $Radius_{EAC}$**<br>$Radius_{Inscribed\ Circle}$ = Radius of the inscribed circle (see left)	NA
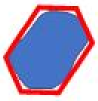	Convex Hull	The perimeter of the convex polygon with the shortest possible perimeter to fully encompass the selection normalized to the EAC	Convex Hull = $Perimeter_{EAC}$***/ $Perimeter_{Convex\ Hull}$<br>$Perimeter_{Convex\ Hull}$ = Perimeter of the convex hull (see left)	NA
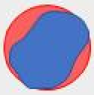	Circularity	The "closeness" of the selection to a perfect circle<br>A circularity of 1 indicates a perfect circle<br>As circularity approaches 0 the selection elongates	Circularity = 4 * π * Area of the Cell / Perimeter of the Cell^2	NA
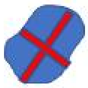	Aspect Ratio	The ratio of the major axis and the minor axis for an ellipse fit to the selection<br>The ellipse of interest is defined as the best fit ellipse centered at the centroid of the selection	Aspect Ratio = Major $Axis_{Selection}$ / Minor $Axis_{Selection}$	NA
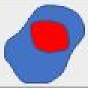	Area of the Nucleus	Area of the nucleus of the selection<br>Nucleus is defined by U-NET/CGAN prediction imaging for DAPI	Area of the Nucleus = Number of pixels contained within the selection * Pixel to Micrometer Conversion Factor for Image	µm
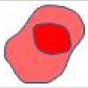	Nucleus to Cell Area Ratio	Ratio of the area of the cell to the area of the nucleus	Cell to Nucleus Area Ratio = Area of the Nucleus / Area of the Cell	NA

*EAC = A circle with an area equal to that of the selection, **$Radius_{EAC}$ = Radius of the EAC, ***$Perimeter_{EAC}$ = Perimeter of the EAC

Figure S11. Shape Metrics. We identified 12 shape metrics that we hypothesized could aptly quantify MSC morphology; This selection was based on previous studies which either categorized MSCs based on morphology [19] or introduced metrics to analyze compactness properties of circles [81]. The selected shape metrics aimed to evaluate either 1) MSC size and/or 2) MSC elongation or roundness and were obtained with ImageJ or Python. The selected shape metrics were area of the cell, perimeter of the cell, proximity, spin index, cohesion, depth index, inscribed circle, convex hull, circularity, aspect ratio, area of the nucleus, and nucleus to cell area ratio.

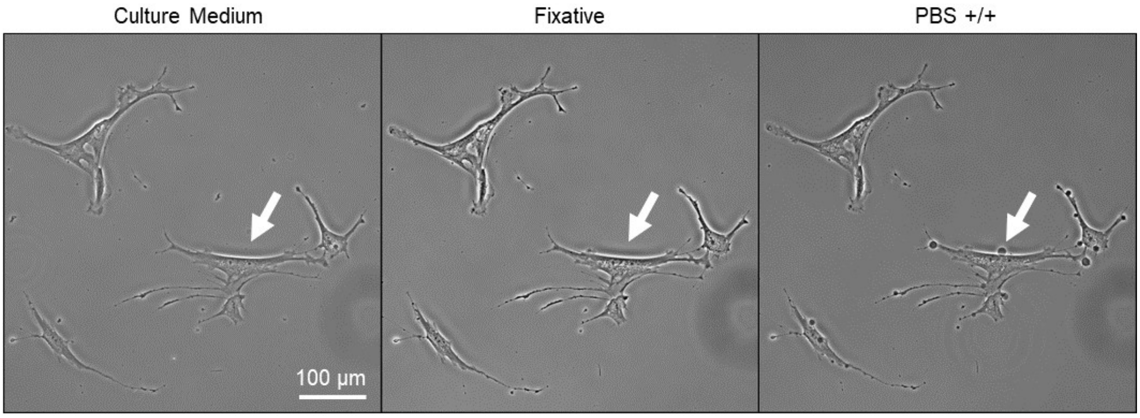

Figure S12. Cell morphology test. To test how the fixative (4% PFA) influenced the cell morphology, we obtained a time series of cell images during the fixation process. Left to right: snapshot of MSCs before (left), during (2 minutes exposure to PFA, middle) and after (15 minutes after PFA fixation, right) the fixation. We found that the morphological change due to PFA fixation was negligible, suggesting minimal impacts on the ML training and predicting accuracy in the time-lapse experiment. While we observed a few small air bubbles (white arrow) near the cell border, they were completely eliminated through washing steps.

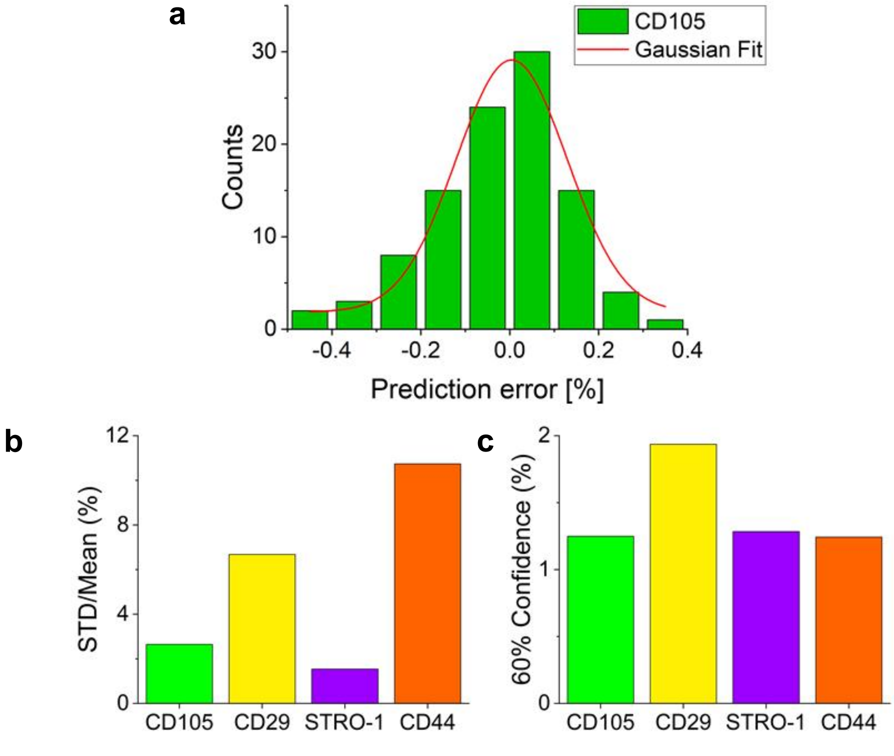

Figure S13. Prediction uncertainty. a. Bar chart demonstrating prediction error distribution of CD105: using the single cell gene-expression comparison data (Fig. S10), we estimated the prediction error used in Fig. 4b. Prediction error was defined as the difference between the target total intensity and the prediction. The calculated value was then normalized by the prediction total intensity value. We found that the prediction error histogram was well described by a Gaussian curve (red curve) for all tested markers. **b.** Bar chart showing the coefficient of variance (CV) for all 4 markers that were studied in the time-lapse experiment. CV is a metric to quantify the variability extent. **c.** Using the data presented in a. we calculated the 60% confidence interval of the prediction error. For all tested markers, we found that this value was less than 2% of the mean intensity.

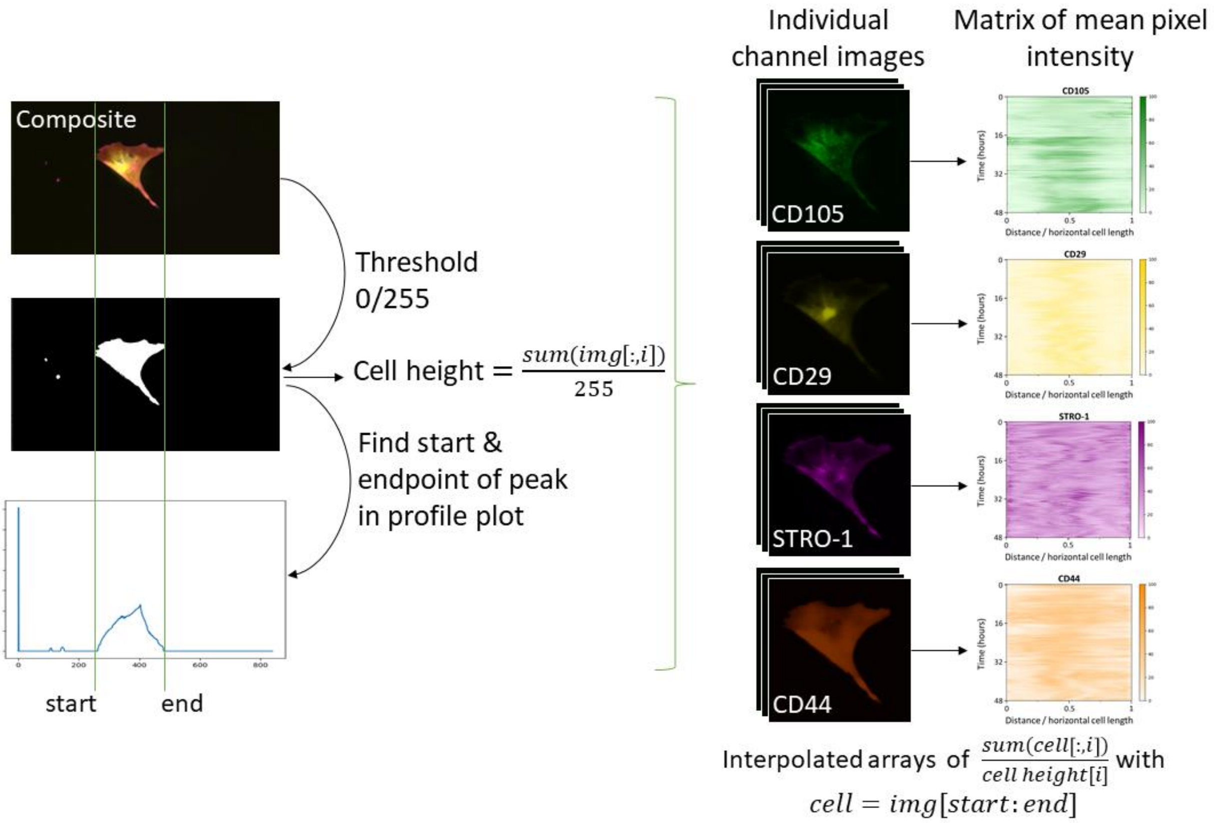

Figure S14. Intracellular fluctuation analysis. Snapshots from Movie S2 were analyzed to extract the mean pixel intensity (averaged over all fluorescent channels) within the cell. With the segmented image (left middle), we determined the left and right edges of the cell, as well as the cell height. These three parameters were then used for calculating the intensity profile projected along the vertical axis (1D horizontal profile). The 1D profile was normalized by the cell height and standardized to have the same length for each timepoint. The final heatmaps, which consist of the computed intensity profiles, show the temporal-spatial intensity fluctuation within a single cell.

Antibody or stain	Source	Catalog #	Host species & reactivity	Concentration
CD105	Invitrogen	MA5-11854	*Mouse / IgG1,*	1:20
CD90	Invitrogen	MA5-32559	*Rabbit / IgG*	1:100
CD73	Invitrogen	MA5-15537	*Mouse / IgG1*	1:100
CD29	Invitrogen	PA5-29606	*Rabbit / IgG*	1:100
CD44	proteintech	15675-1-AP	*Rabbit / IgG*	1:100
CD106	Invitrogen	MA5-11447	*Mouse / IgG1*	1:50
STRO-1	Invitrogen	39-8401	*Mouse / IgM*	1:100
CD146	proteintech	17564-1-AP	*Rabbit / IgG*	1:100
NucBlue	Invitrogen	R37606	-	1 drop / ml

Table S1. Immunostaining reagents.

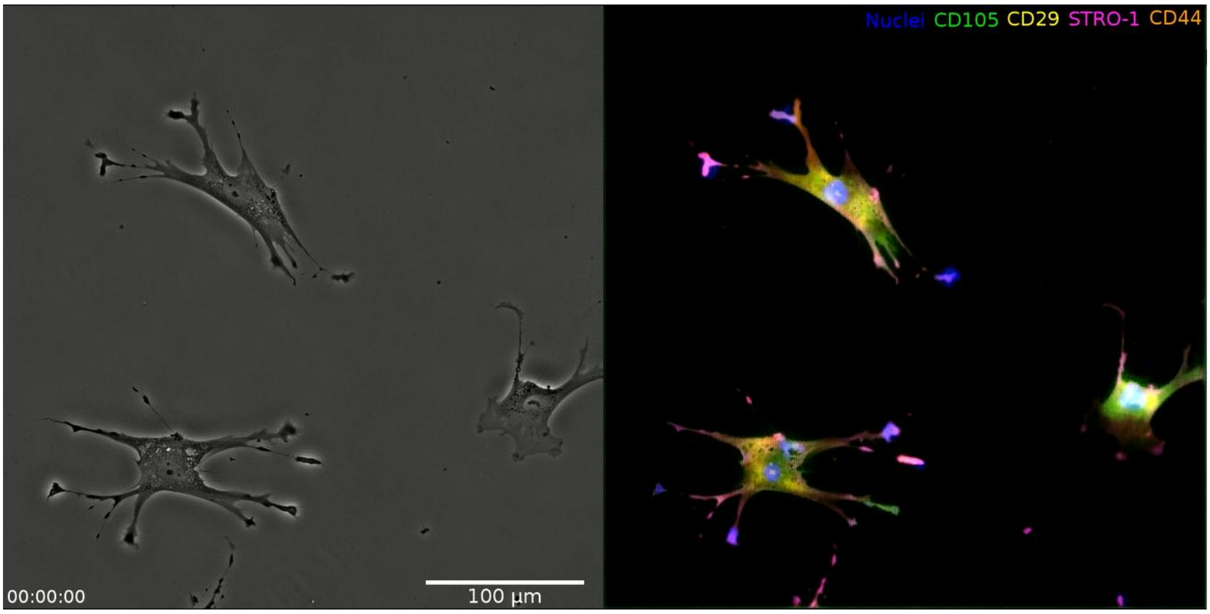

Video S1. ML prediction time-lapse video. Application demonstration that shows our reported AI-based label-free tool can convert phase contrast time-series images into a multi-marker fluorescent video. Phase contrast time-lapse was acquired over 48 hours with a time interval of 2 minutes (left, Methods). Predicted fluorescence video (right) was generated by combining the images predicted by 5 different ML models (Nucleus, CD105, CD29, CD44 and STRO-1).

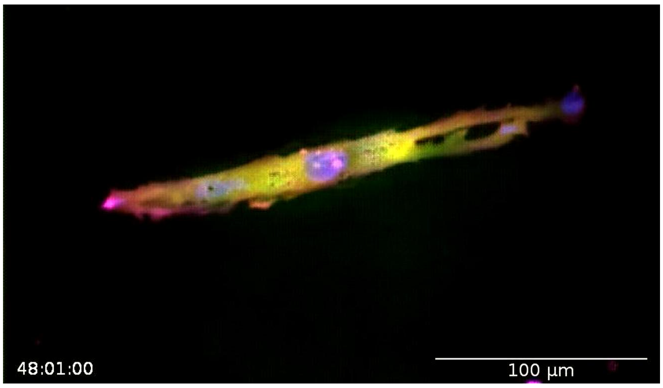

Video S2. Single cell prediction time-lapse video*.* Five channel composite time-lapse video of Nucleus, CD105, CD29, STRO-1 and CD44 consisting of 1341 snapshots (48 hrs). This video was used for the analysis shown in Fig. 4.

References

19. Klinker, M. W., Marklein, R. A., Lo Surdo, J. L., Wei, C.-H. & Bauer, S. R. Morphological features of IFN-γ–stimulated mesenchymal stromal cells predict overall immunosuppressive capacity. Proc. Natl. Acad. Sci.114, E2598 LP – E2607,DOI: 10.1073/pnas.1617933114 (2017)
39. Ronneberger, O., Fischer, P. & Brox, T. U-Net: Convolutional Networks for Biomedical Image Segmentation. InMedicalImage Computing and Computer-Assisted Intervention – MICCAI 2015, 234–241 (Springer International Publishing, Cham,2015).
40. Isola, P., Zhu, J.-Y., Zhou, T. & Efros, A. Image-to-Image Translation with Conditional Adversarial Networks. In2017IEEE Conference on Computer Vision and Pattern Recognition (CVPR), 5967–5976, DOI: 10.1109/CVPR.2017.632 (IEEE,2017).
80. Krizhevsky, A., Sutskever, I. & Hinton, G. ImageNet Classification with Deep Convolutional Neural Networks.Neural Inf.Process. Syst.25, DOI: 10.1145/3065386 (2012).
81. Angel, S., Parent, J. & Civco, D. L. Ten compactness properties of circles: measuring shape in geography.The Can. Geogr./ Le Géographe canadien54, 441–461, DOI: https://doi.org/10.1111/j.1541-0064.2009.00304.x (2010).
